# Pathogenesis of cerebral malformations in human fetuses with meningomyelocele

**DOI:** 10.1186/1743-8454-5-4

**Published:** 2008-03-01

**Authors:** Olga A de Wit, Wilfred FA den Dunnen, Krystyne M Sollie, Rosa Iris Muñoz, Linda C Meiners, Oebele F Brouwer, Esteban M Rodríguez, Deborah A Sival

**Affiliations:** 1 Department of Neurology, University Medical Center, University of Groningen, Hanzeplein 1, 9700 RB, Groningen, The Netherlands; 2 Department of Pathology and Laboratory Medicine, University Medical Center, University of Groningen, Hanzeplein 1, 9700 RB, Groningen, The Netherlands; 3 Department of Obstetrics and Gynecology, University Medical Center, University of Groningen, Hanzeplein 1, 9700 RB, Groningen, The Netherlands; 4 Instituto de Anatomía, Histologíca y Patología Facultad de Medicina, Universidad Austral de Chile, Valdivia, Chile; 5 Department of Radiology, University Medical Center, University of Groningen, Hanzeplein 1, 9700 RB, Groningen, The Netherlands; 6 Department of Pediatrics, University Medical Center, University of Groningen, Hanzeplein 1, 9700 RB, Groningen, The Netherlands

## Abstract

**Background:**

Fetal spina bifida aperta (SBA) is characterized by a spinal meningomyelocele (MMC) and associated with cerebral pathology, such as hydrocephalus and Chiari II malformation. In various animal models, it has been suggested that a loss of ventricular lining (neuroepithelial/ependymal denudation) may trigger cerebral pathology. In fetuses with MMC, little is known about neuroepithelial/ependymal denudation and the initiating pathological events.

The objective of this study was to investigate whether neuroepithelial/ependymal denudation occurs in human fetuses and neonates with MMC, and if so, whether it is associated with the onset of hydrocephalus.

**Methods:**

Seven fetuses and 1 neonate (16–40 week gestational age, GA) with MMC and 6 fetuses with normal cerebral development (22–41 week GA) were included in the study. Identification of fetal MMC and clinical surveillance of fetal head circumference and ventricular width was performed by ultrasound (US). After birth, MMC was confirmed by histology. We characterized hydrocephalus by increased head circumference in association with ventriculomegaly. The median time interval between fetal cerebral ultrasound and fixing tissue for histology was four days.

**Results:**

At 16 weeks GA, we observed neuroepithelial/ependymal denudation in the aqueduct and telencephalon together with sub-cortical heterotopias in absence of hydrocephalus and/or Chiari II malformation. At 21–34 weeks GA, we observed concurrence of aqueductal neuroepithelial/ependymal denudation and progenitor cell loss with the Chiari II malformation, whereas hydrocephalus was absent. At 37–40 weeks GA, neuroepithelial/ependymal denudation coincided with Chiari II malformation and hydrocephalus. Sub-arachnoidal fibrosis at the convexity was absent in all fetuses but present in the neonate.

**Conclusion:**

In fetal SBA, neuroepithelial/ependymal denudation in the telencephalon and the aqueduct can occur before Chiari II malformation and/or hydrocephalus. Since denuded areas cannot re-establish cell function, neuro-developmental consequences could induce permanent cerebral pathology.

## Background

Spina bifida aperta (SBA) is associated with cerebral pathology, such as hydrocephalus, Chiari II malformation, heterotopias and cortical abnormalities [1-3]. In SBA, ventriculomegaly of the posterior horns (i.e. colpocephaly) is already present during fetal life. Because of open communication between the posterior and frontal horns, colpocephaly must reflect an *ex vacuo *phenomenon. However, during the first weeks after birth, high-pressure hydrocephalus is present in the majority of SBA neonates. Little is known about the pathogenesis of these cerebral abnormalities.

According to various animal models, loss of neuroepithelial/ependymal cells is associated with hydrocephalus [4,5]. Analogous to these observations in laboratory animals, Sarnat reported presence of ependymal denudation in human hydrocephalic infants [6]. In these hydrocephalic infants, a positive relationship between the amount of ventricular distension and the area of ependymal denudation was indicated [6]. In accordance with Sarnat, Domínguez-Pinos *et al*. reported presence of ependymal denudation in fetuses with mild communicating hydrocephalus of various origins [7]. However, these fetuses did not show a positive relationship between ventricular dilatation and ependymal denudation [7]. In fetal SBA, information on a potential relationship between hydrocephalus and ependymal denudation is still lacking. Since prenatal surgical closure of the MMC can prevent hydrocephalus and Chiari II malformation [8], insight into such a relationship would be clinically relevant. If hydrocephalus triggers neuroepithelial/ependymal denudation, prevention of hydrocephalus could attenuate loss of neuroepithelial and ependymal cells. Alternatively, neuroepithelial/ependymal denudation and hydrocephalus could share a common etiology. This may involve a primary alteration of the neuroepithelium or ependyma before the initiation of hydrocephalus, as described in hyh mutant mice [9]. In such animal models, the process of neuroepithelial/ependymal denudation is associated with other pathology, such as: abnormalities of junction proteins, abnormal migration of disassembled ependymal cells (into the ventricle and neuropil; i.e. ependymal rosettes), and arrival of numerous macrophages at the site of denudation [4,10,11]. If analogous pathological sequences are involved in human fetuses with MMC, prenatal treatment of hydrocephalus would not prevent neuroepithelial/ependymal denudation. In this respect, investigation of the association between neuroepithelial/ependymal abnormalities and fetal ultrasound (US) measurements (ventriculomegaly/hydrocephaly) may provide new therapeutic insight.

In the present study, we hypothesize that neuroepithelial/ependymal denudation can be initiated before hydrocephalus. If so, this would implicate that both abnormalities can share a common etiology rather than the former being a consequence of the latter. Hence, in this study, we associated longitudinal ultrasound (US) parameters for hydrocephalus with the occurrence of neuroepithelial/ependymal denudation.

## Methods

### Patients

We retrospectively investigated histological sections of eight MMC patients (7 fetuses and 1 neonate) and six fetal controls (collected over the last 15 years at the University Medical Center Groningen). Fetal GA varied from 16–40 weeks. Parents had given informed consent. The medical ethical committee (UMCG, Netherlands) approved retrospective data collection.

In one patient, delivery was caused by *abruptio placentae*. In the other cases, delivery was induced (prostaglandine-E2 medication; n = 4) and/or assisted (cephalocentesis; n = 2 or vacuum extraction; n = 1). After initiation of delivery, birth occurred the same day (median 12 hours; Table 1). All, except one patient (case 7), died during delivery. Case 7 died during the first postnatal week. Fetal controls (n = 6) varied from 22 to 41 weeks GA. Control fetuses had died from: Potter syndrome, bacteraemia, premature labour, maternal diabetes, umbilical cord strangulation, and complicated twin pregnancy. In control fetuses, delivery was initiated spontaneously (n = 4), by caesarean section (n = 1) or induced by prostaglandine-E2 medication (n = 1). In all control fetuses, cerebral malformations were absent.

**Table 1:** Clinical data

Case	GA	MMC	Malformations		Delivery induction	Time	Recent acute bleedings
			cerebrala^a^	non-CNS		induction-delivery	
1	16	C-Th	holopros.; fused basal ganglia; cortical dysplasia	palatoschisis annular pancreas atresia ani scoliose	PG	< 1 day	PF
2	21	L-S	-	reduction defect of extremity	PG	< 1 day	IVH, SAH, PF
3	21	Th-L	-	single umbilical artery	PG	< 1 day	IVH, SAH
4	22	L-S	-	dysplasia costae kyfose	PG	< 0.5 day	PF
5	34	Th-L	-	OEIS complex pes calcaneovalgus lung hypoplasia kyphosis	abruption placentae	ni	IVH
6	37	L-S	-	-	cephalocentesis	< 1 day	IVH, SAH, PF
7	39	L-S	agenesis of: cerebellum, nucleus olivaris inferior, and pontine nuclei	pes calcaneovalgus finger-malformation	VE	ni	IVH, PF
8	40	Th-L	hypoplasia of cerebellum	palatoschisis pes calcaneovalgus ASD	cephalocentesis	< 1 day	IVH, SAH

Legends: GA = gestational age in weeks, MMC = segmental level of the meningomyelocele, ^a^cerebral malformations other than ventriculomegaly and Chiari malformation, holopros. = holoprosencephaly, non-CNS = outside the central nervous system, C = cervical, Th = thoracal, L = lumbar, S = sacral, - = absent, OEIS complex = Omphalocele, Exstrophy, Imperforate anus, Spinal defects, ASD = atrium septum defect, PG = prostaglandin, VE = vacuum extraction, Time induction-delivery = time between induction and delivery, ni = no induction, PF = fossa posterior, IVH = intraventricular bleeding, SAH = subarachnoidal hemorrhage.

### Fetal cerebral ultrasound assessments

Identification of fetal MMC and clinical surveillance of fetal head circumference and ventricular width was performed by US. After birth, MMC was confirmed by histology and serial fetal US assessments were retrospectively studied with special interest in cerebellar growth and localization, ventriculomegaly, macrocephaly (head circumference > 2 SD) and hydrocephalus (macrocephaly in association with ventriculomegaly) [12-15]. Gestational age during US assessment varied from 21–39 weeks. Time between fetal cerebral US assessment and *post mortem *fixation was four days (median duration). The neonate was assessed by magnetic resonance imaging (MRI) at postnatal day 3.

### Histological preparation

Histological preparations in fetal MMC and controls involved identical procedures. In both groups, post-mortem time before fixation ranged from 2 h (most cases) to 3 days. The brain and spinal cord were fixed by immersion in 4% formalin in phosphate buffered saline (pH 7.4). NaCl was added to the fixative to make the tissue float in order to overcome deformities during the fixation period of 2 weeks. Paraffin embedded tissue blocks were sectioned at 5 μm thickness. The spinal cord, brainstem, and cerebellum were sectioned transversely; the cerebrum was sectioned coronally. Several sections were obtained from blocks containing parts of cortex (8/8 patients), basal ganglia and thalamus (7/8; case 5 excluded), cerebellum (8/8), germinal matrix (8/8), aqueduct (7/8; case 5 excluded) and 4^th ^ventricle (7/8; case 1 excluded) and stained with haematoxylin-eosin.

In addition, tissue blocks containing samples of the cerebral aqueduct (7/8; case 5 excluded) were serially cut throughout. From several hundred sections per block, 10 representative series were obtained. We therefore mounted every 20^th ^section on a slide, resulting in a series derived from the whole block. In total, this procedure was performed ten times (i.e. for example for the first series: 1^st^, 21^st^, 41^st ^section and so on; for the second series: 2^nd^, 22^nd^, 42^nd ^section and so on), resulting in ten series. Each series was stained by different histochemical and immunocytochemical procedures allowing mutual comparison.

From a theoretical point of view, absence of ependyma could be caused by ependymal denudation or by an artifact (such as by poor fixation, when the *post mortem *interval is prolonged). In order to discern between the two, we characterized "neuroepithelial/ependymal denudation" as presence of an astroglial reaction (demonstrated by anti-GFAP) confined to the area of neuroepithelial/ependymal denudation. Deformities of the aqueduct were assessed as slit like (i.e. luminal narrowing) and forking (i.e. infolding lined by ependymal cells [16]).

### Immunocytochemistry

The following antibodies were used: (1) anti-nestin, raised in rabbit (Santa Cruz Biotechnology, INC., San Diego, CA, USA), 1:100 dilution. During embryogenesis, nestin is an intermediate filament protein, expressed in neuro-epithelial progenitor cells [17]. Incubation was for 60 min, after antigen retrieval using Tris/HCl, at pH 9.0. Secondary (GaMIgG_1_bio, DAKO, Glostrup, Denmark) and tertiary (Avidin-Biotin complex, DAKO, Glostrup, Denmark) antibodies were used at 1:750 and 1:100 dilution, respectively, for 30 min; (2) anti-glial fibrillary acidic protein (GFAP), raised in rabbit (Santa Cruz Biotechnology, INC., San Diego, CA, USA), 1:1000 dilution; (3) anti-caveolin-1 (neuroepithelial/ependymal marker [18] raised in rabbit, affinity purified (Santa Cruz Biotechnology, INC., San Diego, CA, USA), 1:2000 dilution. Incubation for antibodies 2 and 3 was in a moist chamber for 18 h. Anti-rabbit IgG raised in goat (Sigma, Madrid, Spain) was used at a dilution 1:50 for 1 h. Rabbit PAP (Dako, Carpinteria, CA, USA) was used a 1:75 dilution for 30 min. 3.3'-diaminobenzidine tetrahydrochloride (DAB, Sigma, Madrid, Spain) was used as electron donor. Primary and secondary antibodies were diluted in 0.01 M buffered phosphate saline, pH 7.3, containing 0.1% sodium azide and 0.5% Triton X-100. Omission of incubation in the primary antibody was used as a control for the immunoreaction.

## Results

The cranial border of the MMC was located at cervical (n = 1), thoracic (n = 3) or lumbar (n = 4) segmental levels. One fetus was diagnosed with OEIS complex (omphalocele, exstrophy, imperforate anus, spinal defects; case 5). Malformations other than ventriculomegaly and Chiari II malformation are presented in Table 1.

### Fetal cerebral US assessments (Table 2)

**Table 2:** Relation between cerebral malformations and hydrocephalus

Case	GA	ED	V	*hc > 2*	HC	Chiari II	HemoP/Gliosis	Morphologic alteration aqueduct
1	16	+	-	-	-	-	¶	¶
2	21	+	-	-	-	+	+	f
3	21	-	-	-	-	+	¶	¶
4	22	+	+	-	-	+	+	s
5	34	-	-	-	-	-	¶	¶
6	37	+	+	+	+	+	+	f
7	39	+	-	-	-	IV	+	f
8	40	+	+	+	+	+	+	f; s

Legends: GA = gestational age in weeks, ED = signs of ependymal denudation at the aqueduct or cranial border of the aqueduct, V = ventriculomegaly, *hc *= head circumference, HC = hydrocephalus, Chiari II = Chiari II malformation, HemoP = hemosiderophagocytosis, + = present, - = absent, IV = Chiari IV malformation (absence of the cerebellum), ¶ = serial sections at the aqueduct incomplete, f = forking, s = slit like.

In case one, a fetus of 16 weeks GA, there were no signs of ventriculomegaly or Chiari II malformation. In fetuses of 21–22 weeks GA (3 fetuses), signs of Chiari II malformation (3/3) and ventriculomegaly (1/3) were present. In fetuses of 37–40 weeks GA (3 fetuses), hydrocephalus (2/3) became additionally present. In the fetus with OEIS complex (case 5; 34 weeks GA), ventriculomegaly and Chiari II malformation were absent.

### Neonatal MRI assessment

In the neonate (case 7, 39 weeks GA) that lived for the first postnatal week, neonatal MRI was performed at day 3. In this case, the MRI indicated absence of the cerebellum, a small brainstem and a prominent cisterna magna. This malformation is also referred to as Chiari IV malformation [19], Table 2. At day 3, MRI did not reflect signs for high intracranial pressure (such as signs of trans-ependymal CSF leakage on T2 weighted images or compression of peripheral CSF spaces).

### Histological findings

#### Cerebral aqueduct

**Figure 1:**
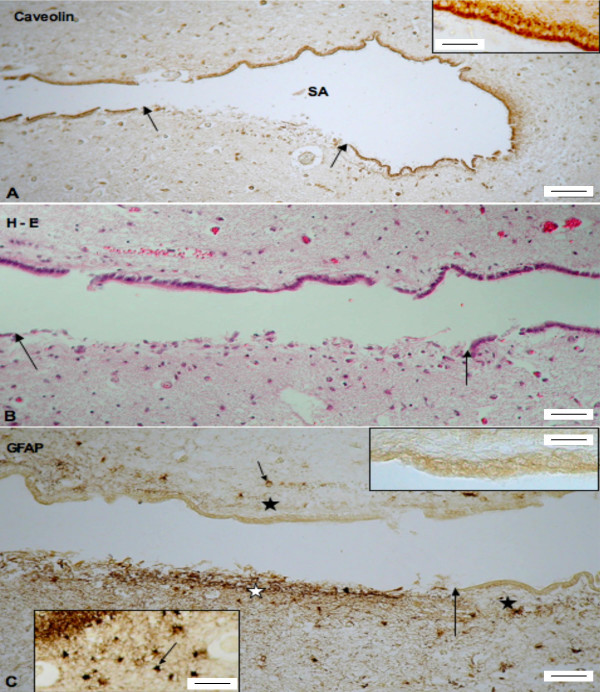
**Ependymal denudation and secondary astroglial reaction at the aqueduct of a fetus with MMC (39 weeks GA)**. A. Transverse section through the aqueduct with immunostaining for caveolin. The ependymal cells are strongly reactive. Arrows indicate an area devoid of ependyma. SA = aqueduct of Sylvius. Scale bar = 100 μm. Insert: Detailed magnification of immature ependyma immunoreacting with anti-caveolin. Scale bar = 20 μm. B. Section adjacent to that of previous figure, stained with haematoxylin-eosin. Arrows indicate an area devoid of ependyma. Scale bar = 50 μm. C. Section adjacent to that of previous figure immunostained with anti-GFAP. Immunoreactive cell processes and cells (astrocytes) are confined to the denuded area (white star). Subependymal neuropil of adjacent areas lined by ependymal cells has only few astrocytes (small arrow) and virtually no astrocyte processes (black stars). The large arrow points to the border of the denuded area. Scale bar = 50 μm. Top insert: Detailed magnification of the non-reactive ependymal and subependymal neuropil. Scale bar = 30 μm. Bottom insert: numerous astrocytes (arrow) in the vicinity of the denuded area. Scale bar = 35 μm.

**Figure 2:**
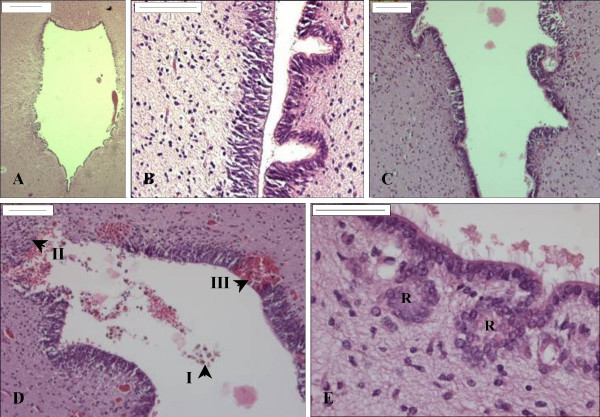
**Haematoxylin-eosin staining of the aqueduct throughout gestation**. A. Transverse section through a wide-open aqueduct of a control fetus (40 weeks GA). Scale bar = 500 μm. B. Transverse section through the aqueduct of a fetus with MMC (22 weeks GA). The lumen appears narrow or slit like. Scale bar = 100 μm. C. Transverse section through the aqueduct of a fetus with MMC (21 weeks GA). Several infoldings are present (i.e. forking). Scale bar = 100 μm. D. Secondary damage at the aqueduct of a fetus with MMC (21 weeks GA). Arrowheads indicate haemosiderophages (I), gliosis (II), and recent bleeding (III). Scale bar = 100 μm. E. Transverse section through the aqueduct of a fetus with MMC (37 weeks GA). The figure shows sub-ventricular rosette formation (R). Scale bar = 50 μm.

**Figure 3:**
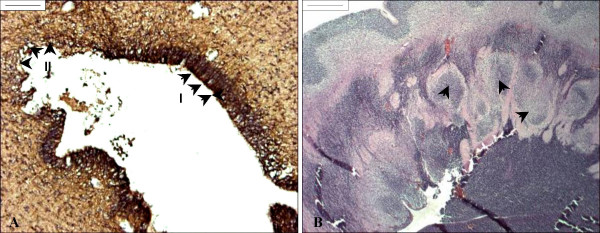
**Ependymal denudation and subcortical heterotopias**. A. Nestin staining of the aqueduct of a SBA fetus of 21 weeks GA. Arrowheads indicate intact (I) and denuded (II) ependymal lining. At the denuded area, the figure shows reduction of nestin-positive cells (brown; DAB) indicative of progenitor cell loss. Scale bar = 100 μm. B. Haematoxylin-eosin staining of the telencephalon of a fetus with MMC (16 weeks GA). Arrowheads indicate subcortical heterotopias associated with ependymal denudation of the lateral ventricle. Scale bar = 500 μm.

The cerebral aqueduct of young fetuses (21, 21 weeks GA) was lined by a multilayered neuroepithelium whereas the aqueduct of older fetuses (27, 39 and 40 weeks GA) was lined by immature ependyma. Both neuroepithelium and ependyma reacted strongly with anti-caveolin (Fig. 1A). In six of eight fetuses (16–40 weeks GA), neuroepithelial/ependymal denudation (characterized by astrogliosis) was present (Figs. 1A–C). The denuded area ranged from a few ependymal cells up to a few millimeters in length (Figs. 1A–C, 2D, 3A). Several denuded areas could be found throughout the series of sections of the aqueduct. The number of denuded areas varied between cases. Additionally, histological signs for hemosiderophagocytosis were present (3/6) (Fig. 2D). In five cases, sections of the aqueduct were complete for morphological assessment. In these cases, ependymal denudation concurred with malformations of the aqueduct (such as forking (4/5), slit-like deformities (2/5) and sub-ependymal rosette formation (cases 6 and 8, 37–40 weeks GA), see Figs. 2A–E, 3A.

#### Telencephalon

At the telencephalon, neuroepithelial/ependymal denudation seemed associated with sub-ventricular heterotopias (16 week GA, Fig. 3B).

The individual relationship between US findings and histological assessments is indicated in Table 2. In all control fetuses, this pathology (ependymal denudation, sub-ventricular heterotopias, gliosis and deformities) was absent.

#### Cerebral hemorrhage

**Figure 4:**
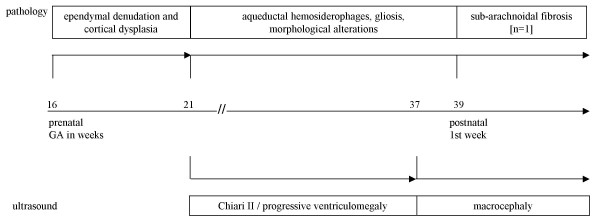
**Histological and ultrasound data in pre- and postnatal SBA according to gestational age**. Histological and US findings are illustrated in chronological order, according to gestational (GA). The time axis is in the middle part of the figure. Histological data of aqueduct and convexity are indicated at the upper part of the figure. US data of ventricular size (Chiari II malformation, ventriculomegaly and macrocephaly) are indicated at the lower part of the figure. During the first half of gestation, neuroepithelial/ependymal denudation is observed before the onset of Chiari II malformation and hydrocephalus.

In all SBA fetuses, recent delivery-related cerebral bleedings were indicated by the presence of numerous (fresh) erythrocytes. These hemorrhages were observed at intra-ventricular (6/7), sub-arachnoidal (4/7) and intra-parenchymal locations (Table I). In the available histological preparations of the convexity, sub-arachnoidal fibrosis was absent from all succumbed SBA fetuses (n = 7), whereas it was present in the neonate that survived for the first postnatal week (case 7). The time relationship between histological and radiological assessments in SBA patients is shown in figure 4. In control fetuses, recent cerebral hemorrhage was present in two out of six fetuses, and sub-arachnoidal fibrosis was absent.

The study also included parts of basal ganglia/thalamus, cerebellum and 4^th ^ventricle (in 7/8, 8/8 and 7/8 cases; respectively). Neuro-pathological findings at these regions were: abnormal fusion or hemorrhages at basal ganglia/thalamus (cases 1 and 7, respectively); cerebellar a- or hypoplasia (cases 7 and 8, respectively) and hemorrhages in the 4^th ^ventricle (cases 2, 3, 5, 6 and 8). In case 3, there was a hemorrhage at the choroid plexus.

## Discussion

In perinatal SBA, we associated the initiation of cerebral pathology with the concurrence of neuroepithelial/ependymal denudation at the aqueduct. In contrast to the controls, fetuses with MMC displayed neuroepithelial/ependymal denudation at the aqueduct. Our data indicate that neuroepithelial/ependymal denudation may precede hydrocephalus and Chiari II malformation.

Most ependymal cell lineages are born and mature at fixed stages of fetal development [5,20]. After closure of the neural tube, choroid plexus villi start to produce cerebrospinal fluid (CSF) [21]. Studies in animal models and humans have shown that this CSF contains essential molecules for neural proliferation and migration [22-27], which is predominantly secreted by choroid plexus and sub-commissural organ [28]. Additionally, fetal ependymal cells can release molecules via their long basal ependymal processes that extend into germinal matrix and white matter. These processes may have a trophic function and provide axonal guidance [5]. Once secreted into CSF, molecules follow the CSF flow, reach distant subventricular zones and, through the subarachnoidal space, the external surface of the developing cerebral cortex [28,29]. This CSF flow is maintained by hydrostatic pressure, arterial pulsations and beating of ependymal cilia [30,31]. When ependymal cells are damaged, functional restoration does not occur [20]. In a previous study, we have indicated that CSF growth factor concentrations (TGF-β) are comparatively low in neonatal hydrocephalic SBA [27]. Additionally, other proteins (such as vimentin) appear over-expressed in Chiari II malformation and aqueduct stenosis [32,33]. These altered concentrations of signaling molecules may influence cerebral development [3].

In fetuses with communicating hydrocephalus, ependymal denudation and/or damage has been described as a consequence of ventricular distension (hydrocephalus) [6]. If such a causative relationship also exists in fetuses with MMC, closure of the MMC may prevent both hydrocephalus and the negative consequences of ependymal denudation. However, our present data indicate that neuroepithelial/ependymal denudation can occur before the onset of hydrocephalus. This is in agreement with the concurrence of ependymal denudation with neural migration disorders in absence of hydrocephalus (i.e. lissencephaly and pachygyria) [34].

The presence of neuroepithelial/ependymal denudation implies loss of progenitor cells. A likely fate of these cells is the CSF, as suggested by findings in human hydrocephalic fetuses [7] and human fetuses with MMC [35]. In accordance with cellular loss into CSF, it has been shown that progenitor cells can be harvested from CSF in preterm hydrocephalic infants [36]. As a consequence, neural proliferation, migration [37] and corticogenesis [5] may be consecutively impaired. Accordingly, we observed cellular heterotopias and abnormal neural migration (at the germinal matrix, n = 1, and cortex, n = 2). For further substantiation, more extensive studies of the lateral ventricles and cerebral cortex of fetuses with MMC are required.

Previous studies have described macrophage invasion and astrogliosis at the denuded areas [7,10]. Accordingly, we also observed invasion of macrophages and astrogliosis in human fetal MMC (figures 1, 2, 3, and 4). Subsequent gliosis and scarring of denuded areas may result in aggregates of ependymal cells in the subventricular zone and lead to the formation of subependymal rosettes [5,38-40].

With ongoing gestation, fetal US recordings indicated concurrence of Chiari II malformation and hydrocephalus. Especially during delivery, these malformations are associated with enhanced risk for venous compression (at the large sinuses) and cerebral hemorrhage [41]. In all our patients with MMC, we observed fresh erythrocytes, which suggest recent, delivery-related cerebral hemorrhages. In accordance with a recent onset of these hemorrhages, fetal arachnoidal fibrosis (at the convexity) was absent in all (n = 7) fetuses with MMC, whereas it was present in the neonate with MMC (n = 1). In this patient, clinical history did not reveal another cause for arachnoidal fibrosis. These data may implicate that prenatal presence of progressive ventriculomegaly (and/or hydrocephalus) in fetal SBA is not related to (fibrosis-induced) malabsorption at the convexity.

## Conclusion

The present investigation on human fetal SBA indicates that neuroepithelial/ependymal denudation and abnormal neural migration can occur before the onset of hydrocephalus and Chiari II malformation. Since denuded areas do not re-establish function, fetal SBA surgery is unlikely to prevent these abnormalities.

## Abbreviations

CSF: Cerebrospinal fluid; DAB: Diaminobenzidine substrate; GA: Gestational age; MMC: Meningomyelocele; MRI: Magnetic resonance imaging; SBA: Spina bifida aperta; TGFβ-1: Transforming growth factor beta-1; US: Ultrasound.

## Competing interests

The authors declare that they have no competing interests.

## Authors' contributions

All authors have read and approved the final version of the manuscript.

OdW: contributed to the study of the sections and preparation of the manuscript.

WdD: contributed to the study design, prepared sections and staining, histological assessments and preparation of the manuscript. KMS: contributed to the collection of the former obstetric assessments and advised during the preparation of the manuscript. RIM: contributed to the serial sections of tissue blocks, immunostaining and interpretation of histological assessments. LM: contributed to radiological assessments and advised during the preparation of the manuscript. OFB: contributed to clinical neurological data collection and advised during the preparation of the manuscript. EMR: contributed to the serial sections of tissue blocks, immunostaining, histological assessments and preparation of the manuscript. DAS: contributed to the study design, histological assessments and preparation of the manuscript.
